# Potential of exogenously sourced kinetin in protecting *Solanum lycopersicum* from NaCl-induced oxidative stress through up-regulation of the antioxidant system, ascorbate-glutathione cycle and glyoxalase system

**DOI:** 10.1371/journal.pone.0202175

**Published:** 2018-09-04

**Authors:** Mohammad Abass Ahanger, Mohammed Nasser Alyemeni, Leonard Wijaya, Saud A. Alamri, Pravej Alam, Muhammad Ashraf, Parvaiz Ahmad

**Affiliations:** 1 Department of Botany, Govt Post-Graduation College Rajouri, Jammu and Kashmir, India; 2 Botany and Microbiology Department, College of Science, King Saud University, Riyadh, Saudi Arabia; 3 Biology Department, College of Science and Humanities, Prince Sattam bin Abdulaziz University, Alkharj, Kingdom of Saudi Arabia (KSA); 4 International Centre for Chemical and Biological Sciences, University of Karachi, Pakistan; 5 Department of Botany, S.P. College, Srinagar, Jammu and Kashmir, India; Huazhong Agriculture University, CHINA

## Abstract

The protective role of exogenously applied kinetin (10 μM KN, a cytokinin) against the adverse effects caused by NaCl-induced (150 mM) stress in *Solanum lycopersicum* was investigated. Application of KN significantly enhanced growth and biomass production of normally grown plants (non-stressed) and also mitigated the adverse effect of NaCl on stressed plants to a considerable extent. Among the examined parameters, chlorophyll and carotenoid contents, photosynthetic parameters, components of the antioxidant system (both enzymatic and non-enzymatic), osmotica accumulation, and mineral uptake exhibited a significant increase following the application of KN. Furthermore, KN application reduced the generation of reactive free radical hydrogen peroxide, coupled with a significant reduction in lipid peroxidation and an increase in membrane stability. The activities of antioxidant enzymes, and glyoxylase system were found to be promoted in plants exposed to NaCl, and the activities were further promoted by KN application, thereby protecting *S*. *lycopersicum* plants against NaCl-induced oxidative damage. Further strengthening of the antioxidant system in KN supplied plants was ascribed to regulation of ascorbate-glutathione cycle, phenols and flavonoids in them. The levels of proline and glycine betaine increased considerably in KN-treated plants, thereby maintaining relative water content. Moreover, exogenous KN application reduced the inhibitory effects of NaCl on K^+^ and Ca^2+^ uptake, which resulted in a considerable reduction in tissue Na^+^/K^+^ ratio.

## Introduction

Salinity-induced decline in growth and development of plants occurs due to many factors, resulting in cumulative effects on key physiological processes, such as disruption in ion homeostasis leading to altered water balance, and inhibition of photosynthesis and other biochemical processes, including enzymes’ activity [1–3]. However, to counteract the negative effects of environmental extremes and maintain efficient metabolism, plants employ several tolerance mechanisms, including: (a) accumulation of compatible organic osmolytes, so that water uptake and the associated physiological pathways are least affected; (b) efficient partitioning and compartmentalization of toxic levels of ions into less sensitive tissues or organelles, such as the vacuole; and (c) up-regulation of the antioxidant system for rapid neutralization of stress-generated toxic ROS [3–6]. High salt (NaCl)concentrations are believed to induce both osmotic and ionic stresses. Among the osmolytes, low molecular weight solutes such as glycine betaine (GB), proline, amino acids, and soluble sugars have been reported to contribute effectively to osmotic adjustment and protection against osmotic stress [7,8]. The roles of these osmolytes in cellular osmotic adjustment, stabilization of membrane integrity, and the detoxification of toxic ions during salt stress have been well reported [9]. Accumulation of osmolytes has been observed to protect polypeptide dissociation and preserve the photosynthetic oxygen-evolving complex [10] as well as the activity of ribulose-1, 5-bisphosphate carboxylase (Rubisco) during NaCl-induced stress [1]. Among the antioxidants (both enzymatic and non-enzymatic), superoxide dismutase (SOD), catalase (CAT), peroxidases and reductases [including ascorbate peroxidase (APX) and glutathione reductase (GR)], ascorbic acid (AsA), and glutathione (GSH and GSSG) are the key candidates which contribute to stress tolerance by mediating protection of major physiological and biochemical processes [5]. Antioxidants mediate protection through their involvement in maintaining redox buffering for the optimal functioning of major pathways, including photosynthetic electron transport and enzyme activity [2,3,5,11]. However, for the elimination of toxic methylglyoxal glyoxylase system constituted of two key enzymatic components is up-regulated [12]. Thus, each antioxidant plays its specific role in counteracting a specific type of stress generated ROS. The biggest challenge before the scientists is to enhance the crop production under stressful environment. To achieve this goal. External supplementation of phytohormones could be a sustainable approach under for crop production under salinity.

Plant growth hormones have also been reported to play key roles in mediating several responses during stressful cues for growth and developmental regulation [2]. Recently, KN has been reported to enhance salt tolerance in soybean through its interaction with other growth hormones [13]. Exogenous application of plant growth hormones, such as cytokinins, has been shown to enhance the yield potential of agricultural crops under field conditions [14–16]. Cytokinins are believed to stimulate processes such as water uptake and cell division and enhance the synthesis of chlorophylls and organ development, leading to rapid regeneration and proliferation of shoot tissues [17,18]. KN is a synthesized form of artificial cytokinins and has been reported to ameliorate growth inhibition in several crop plants exposed to stress, such as waterlogging [19], salinity [20], and metal stress [21]. Exogenous application of KN can alleviate toxic effects on growth and photosynthesis by modulating the endogenous profile of other growth hormones, including salicylic acid, gibberellic acid, and jasmonic acid, whereas it acts antagonistically against others such as abscisic acid [13]. However, the KN-induced stress tolerance mechanism has received little attention, including KN-mediated salt exclusion and the restricted absorption from soil solution and subsequent distribution within the tissues or compartmentalization into vacuoles [22].

Tomato (*Solanum lycopersicum* L.) is one of the largest commercially consumed vegetables. It has high contents of bioactive compounds, such as β-carotene, lycopene, flavonoids, and ascorbic acid, and has accordingly been considered as an effective anti-oxidative and anti-cancerous fruit [23]. In the present study, we aimed to evaluate whether or not application of KN enhances antioxidant metabolism and osmoregulation in *S*. *lycopersicum*, plants and whether this can reverse the adverse effects of salt stress on growth, pigment synthesis, and ion accumulation.

## Material and methods

Tomato seeds (var. Pusa Rohini) were obtained from Indian Agricultural Research Institute, Pusa, New Delhi, India. The seeds were surface sterilized using NaOCl (5%) and allowed to germinate in Petri plates lined with blotting paper wetted with distilled water. After germination, healthy seedlings were transplanted into plastic pots of 20-cm diameter and filled with reconstituted sand supplemented with vermicompost and were supplemented with full-strength Hoagland’s nutrient solution (200 mL) on every alternate day for 10 days. For inducing salinity stress modified Hoagland’s nutrient solution supplemented with NaCl (150 mM) was used for another 20 days and 10 μM KN (20 mL per pot) was sprayed foliarly using Teepol (0.1%) as surfactant. During the entire growth period pots were kept under green house with natural climatic conditions having day/night temperatures of 26 /16°C, relative humidity of 70–75% and an average photoperiod of 18 hrs light/6 hrs night. After 30 days of growth plants were uprooted carefully and analyzed for different parameters following standard protocols described hereunder.

### Plant length and dry weight

Plant length was measured after uprooting using a manual scale. Dry weight was recorded after oven-drying the tissue at 70°C for 24 h.

### Estimation of photosynthetic pigments

Fresh leaf tissues were extracted in acetone (80%) using pestle and mortar. Optical density of the supernatant was measured spectrophotometrically at 480, 645, and 663 nm [24].

### Estimation of chlorophyll fluorescence and gas exchange parameters

Chlorophyll fluorescence parameters were measured in fully expanded leaves using PAM chlorophyll fluorimeter (H. Walz, Effeltrich, Germany) [25]. Measurement of CO_2_ assimilation rate (*A*), stomatal conductance (*gs*) and transpiration rate (*E*) was carried in uppermost fully expanded leaves using infrared gas analyzer (LCA-4 model, Analytical Development Company, Hoddesdon, England).

### Determination of leaf relative water content (RWC)

Method described by Smart and Bingham [26]was employed for the determination of RWC and calculation was done using following formula:

$$\text{RWC}=\frac{\text{Fresh weight}-\text{Dry weight}}{\text{Turgid weight}-\text{Dry weight}}\text{ X }100$$ (1)

### Estimation of hydrogen peroxide (H_2_O_2_), lipid peroxidation (MDA) and electrolyte leakage

For estimation of content of hydrogen peroxide (H_2_O_2_) spectrophotometric method described by Velikova et al. [27] was followed and absorbance was measured at 390 nm. Standard curve of H_2_O_2_ was used for calculation and expressed as nM g^-1^ FW.

Lipid peroxidation was estimated in terms of formation of malondialdehyde content and extinction coefficient of 155 mM^-1^cm^-1^ was used for calculation [28].

Electrolyte leakage was determined using the method of Dionisio-Sese and Tobita [29]and formula employed for computation was:

$$\text{Electrolyte leakage}\left(\%\right)=\left(\text{E}{\text{C}}_{1}-\text{E}{\text{C}}_{0}\right)/\left(\text{E}{\text{C}}_{2}-\text{E}{\text{C}}_{0}\right)\times 100$$ (2)

### Determination of antioxidant enzyme activities and ascorbate-glutathione cycle

For extraction of antioxidant enzymes 0.5 g of fresh leaf tissue was macerated in ice-cold potassium phosphate buffer (100 mM, pH 7.0) containing PVP (1%) in a pre-chilled mortar and pestle. The homogenate was centrifuged at 12000 × *g* for 15 min at 4°C and supernatant was used as an enzyme source for assay of SOD, CAT, APX, and GR.

Superoxide dismutase (SOD, EC1.15.1.1) activity was estimated by measuring its ability to inhibit the photochemical reduction of nitroblue tetrazolium (NBT) at 560 nm. Assay mixture containing 100 mM phosphate buffer (pH 7.4), methionine (10 mM), EDTA (1.0 mM), riboflavin (50 μM), NBT (75 μM) and 100 μL enzyme extract was incubated under fluorescent light for 15 min [30]. Activity was expressed as U mg^-1^ protein.

For assaying activity of catalase (CAT, EC1.11.1.6) decomposition of H_2_O_2_ was monitored at 240 nm for 2 min. Reaction mixture (2 mL) contained 50 mM phosphate buffer (pH 6.0), 0.1 mM EDTA, 20 mM H_2_O_2_, and 100 μL enzyme extract. Extinction coefficient of 39.4 mM^-1^cm^-1^ was used and activity was expressed as U mg^-1^ protein [31].

Glutathione *S*-transferase (GST, EC: 2.5.1.18) was estimated according to the procedure of Hasanuzzaman and Fujita [32] and change in absorbance was read spectrophotometrically at 340 nm for 2 min. The activity of GST was calculated using the extinction coefficient of 9.6 mM^−1^ cm^−1^.

For determination of APX (APX, EC1.11.1.1) activity, 2 mL reaction mixture containing 50 mM phosphate buffer (pH 7.5), 0.1 mM EDTA, 0.3 mM ascorbate, 100 μL enzyme extract and 100 μL H_2_O_2_ was monitored for 2 min at 290 nm [33]. For calculation molar extinction coefficient of 2.8 mM^-1^ cm^-1^ was used and activity was expressed as U mg^-1^protein.

Glutathione reductase (GR, EC1.6.4.2) was assayed by measuring the change in absorbance at 340 nm for 3 min. Reaction mixture contained potassium phosphate buffer (100 mM; pH 7.0), EDTA (1.0 mM), NADPH (50 μM), oxidized glutathione (100 μM) and 100 μL enzyme in a final volume of 3 mL. Activity was expressed as U mg^-1^ protein [34].

Methods of Miyake and Asada [35]and Nakano and Asada [33] were employed for the estimation of monodehydroascorbate reductase (MDHAR, EC1.6.5.4) and dehydroascorbate reductase (EC:1.8.5.1) activities. The MDHAR activity was expressed as nmol NADPH oxidized / (EUmg^−1^ protein). The DHAR activity was determined by monitoring change in absorbance at 265 nm for 1 min with a spectrophotometer (Beckman 640D, USA) using an extinction coefficient of 14 mM^−1^ cm^−1^.

Ascorbate was estimated in fresh leaves after homogenizing in ice-cold meta-phosphoric acid (5%) containing EDTA (1 mM). Homogenate was centrifuged at 10,000 rpm for 20 min and the supernatant was used for the estimation of ascorbate [36].

The glutathione pool was determined using the method of Yu et al. [37] with slight modifications [38]. Standard curves with known concentrations of GSH and GSSG were used for calculations.

### Estimation of glyoxalase system

For determination of glyoxalase I (EC: 4.4.1.5) and glyoxylase II (EC: 3.1.2.6) method described by Hasanuzzaman and Fujita [32] was followed. Assay mixture for glyoxylase I contained K-phosphate buffer (100 mM, pH 7.0), magnesium sulphate (15 mM), GSH (1.7 mM) and methylglyoxal (3.5 mM) in a final volume of 900 μL. The change in absorbance was recorded at 240 nm after adding methylglyoxal. For glyoxylase II, the reaction mixture (1.5 mL) contained 100 mM Tris–HCl buffer (pH 7.2), DTNB (0.2 mM) and 1 mM *S*-D-lactoylglutathione. Extinction coefficients of 3.37 mM^-1^ cm^-1^ and 13.6 mM^-1^ cm^-1^ were used for the calculation of glyoxylase I and II, respectively.

### Total phenols and flavonoids

Plant samples were extracted in methanol and centrifuged at 10,000 rpm for 10 min. Total phenolic content was determined by the Folin-Ciocalteu reagent using the method of Chun et al. [39] and expressed as mg gallic acid equivalent (GAE) g^−1^ of extract (mg g^−1^). For estimation of flavonoid content method of Zhishen et al. [40] was followed using catechin as standard. Absorbance was recorded at 510 nm using a spectrophotometer flavonoid content was expressed as mg catechin equivalents g^−1^ of extract (mg g^−1^).

### Estimation of proline and glycine betaine

Proline was extracted using toluene and the absorbance was read at 520 nm [41]. Standard curve of proline was used for calculation and content was expressed as μmol proline g^-1^ fresh weight.

The method described by Grieve and Grattan [42] was adopted for the estimation of glycine betaine, and formation of periodide crystals was read at 365 nm after reacting the mixture with cold KI–I_2_ reagent under acidic conditions.

### Determination of tissue Na, K, and Ca

Dry plant samples (0.5 g) were acid-digested using sulfuric acid and nitric acid (1:5, v/v) at 60°C. The digested samples were cooled and read in a flame photometer for the estimation of Na, K, and Ca.

### Statistical analysis

Data were presented as the means of three replicates with ±SE calculated. The data were subjected to a statistical analysis using analysis of variance for a completely randomized design, followed by the Duncan’s multiple range test, and least significant difference (LSD) calculated at 0.05%.

## Results

### Growth and biomass yield

**Fig 1:**
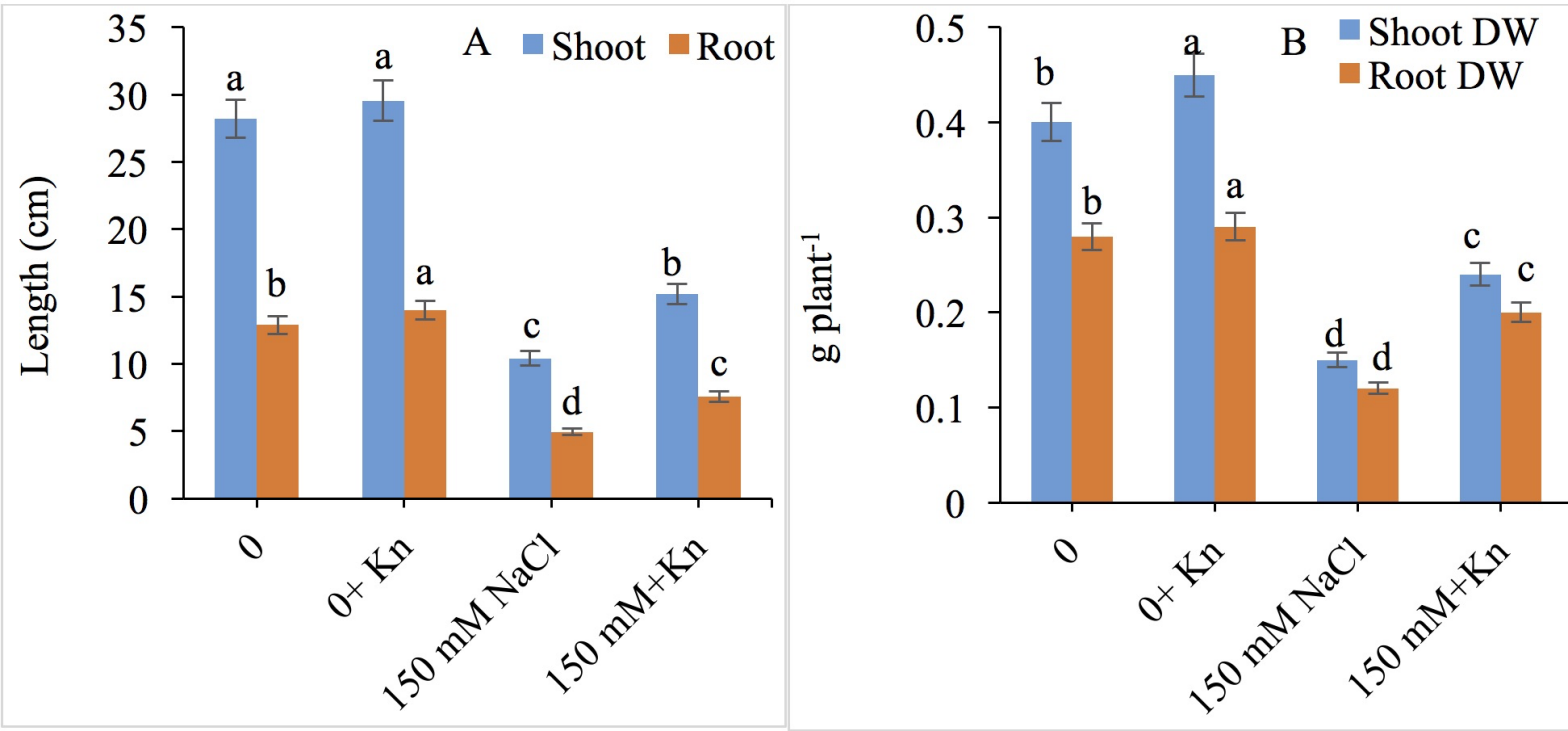
Ameliorating role of kinetin (Kn) on length and dry weight of shoot and root under NaCl toxicity in tomato. Data presented are the means ± SE (n = 3). Different letters indicate significant difference at P ≤ 0.05.

The tomato plants treated with NaCl (150 mM) exhibited a significant reduction in all growth parameters measured. For example, shoot and root lengths decreased by 63.11% and 61.64%, respectively, and shoot and root dry weights by 62.50% and 57.14%, respectively, compared with the control. Exogenous application of KN (10 μM) to tomato plants enhanced shoot length by 45.91% and root length by 52.83% compared with those of salt stressed plants alone. However, KN application to NaCl-treated plants (150 mM NaCl + KN) improved the shoot and root dry weights by 60.00% and 66.66%, respectively over NaCl treated plants alone (Fig 1A and 1B).

### Pigment content

Relative to the control, NaCl treatment reduced the synthesis of chlorophyll *a*, chlorophyll *b*, total chlorophyll, and carotenoids by 52.12%, 39.58%, 47.88%, and 40.00%, respectively over control. However, exogenously applied KN mitigated the negative effects of NaCl by enhancing the synthesis of chlorophyll *a* by 22.22%, chlorophyll *b* by 31.03%, total chlorophyll by 25.67%, and carotenoids by 9.80% compared with those of the NaCl-stressed plants alone (Table 1).

**Table 1:** Exogenous kinetin (Kn) enhanced the pigment (mg g^-1^ FW) content in tomato under NaCl toxicity. Data presented are the means ± SE (n = 3). Different letters indicate significant difference at P ≤ 0.05.

Treatments	Chl a	Chl b	Total Chl	Carotenoids
**0**	0.94±0.021b	0.48±0.006a	1.42±0.091b	0.85±0.066b
**0+ Kn**	0.99±0.026a	0.49±0.006a	1.47±0.097a	0.98±0.075a
**150 mM NaCl**	0.45±0.005d	0.29±0.002c	0.74±0.056d	0.51±0.031d
**150 mM +Kn**	0.55±0.008c	0.38±0.004b	0.93±0.072c	0.56±0.045c

### Chlorophyll fluorescence

Table 2 represents the effect of NaCl and KN on chlorophyll fluorescence. NaCl stress declines the Fv/Fm by 18.82%, ϕPSII by 13.84%, ϕ_exc_ by 28.81% and qP by 27.47% but it enhanced the NPQ by 20.68% compared to control plants. External supplementation of KN restored the chlorophyll fluorescence by enhancing Fv/Fm, ϕPSII, ϕ_exc_ and qP by 42.02%, 57.14%, 23.19% and 24.24% respectively over the plants treated with NaCl alone. NPQ showed decline in KN supplied NaCl stressed plants by 26.31% over NaCl treated plants alone.

**Table 2:** Effect of kinetin (Kn) on chlorophyll fluorescence in tomato under NaCl toxicity. Data presented are the means ± SE (n = 3). Different letters indicate significant difference at P ≤ 0.05.

Treatments	Efficiency of PSII (Fv/Fm)	Quantum yield of PSII (ϕPSII)	Capture efficiency of PSII (ϕ_exc_)	Photochemical quenching (qP)	Non-photochemical quenching (NPQ)
**0**	0.85±0.06b	0.65±0.03c	0.59±0.007b	0.91±0.08a	0.58±0.007c
**0+ Kn**	0.95±0.09a	0.72±0.05b	0.63±0.02a	0.94±0.09a	0.40±0.002d
**150 mM NaCl**	0.69±0.02c	0.56±0.005d	0.42±0.002d	0.66±0.01c	0.95±0.08a
**150 mM +Kn**	0.98±0.12a	0.88±0.08a	0.53±0.004c	0.82±0.05b	0.70±0.05b

### Gas exchange parameters

**Fig 2:**
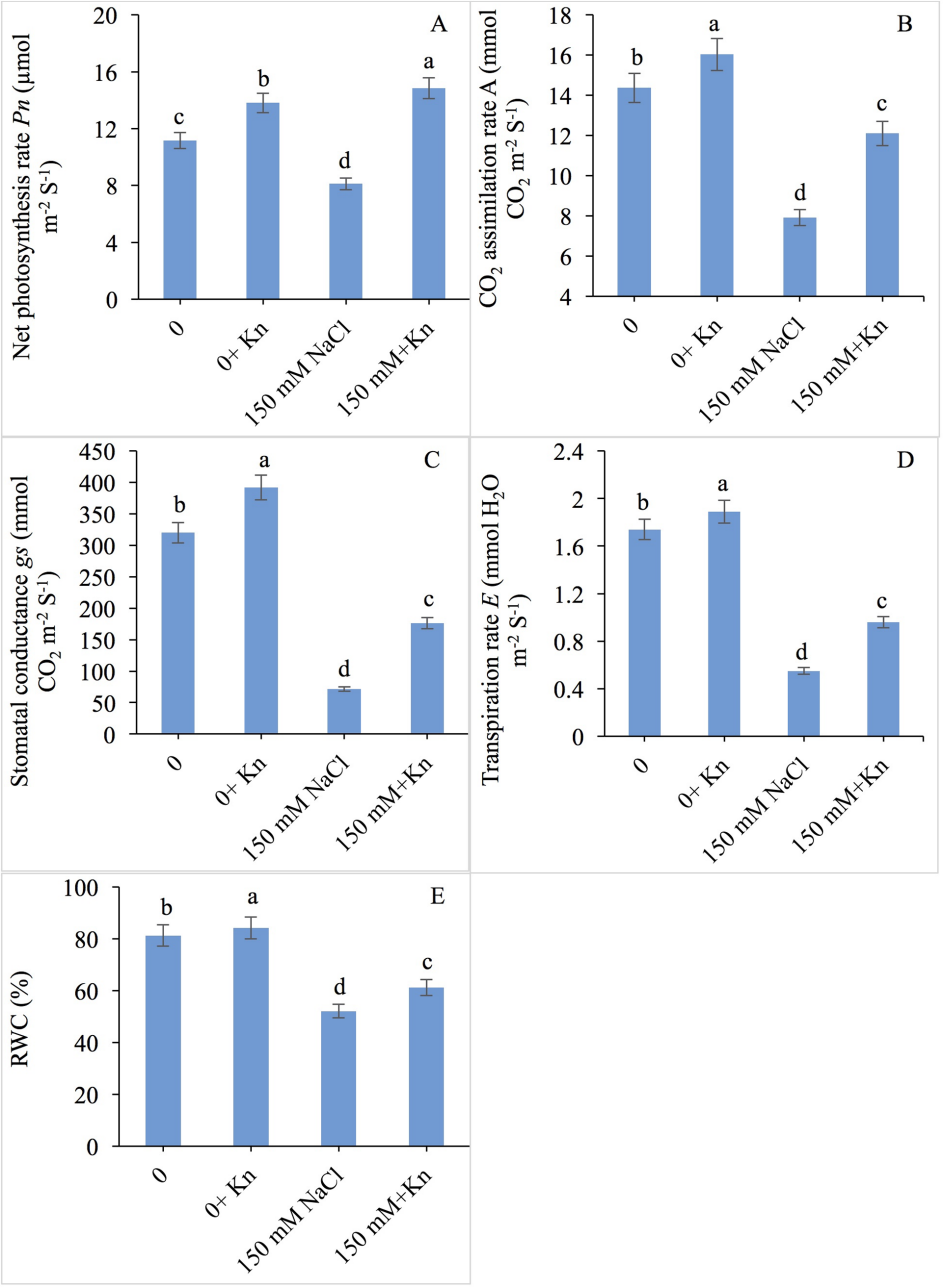
Effect of kinetin (Kn) on gas exchange parameters and relative water content in tomato under NaCl toxicity. Data presented are the means ± SE (n = 3). Different letters indicate significant difference at P ≤ 0.05.

The NaCl treated plants showed decrease in *Pn*, *A*, *gs* and *E* by 27.17%, 44.95%, 77.07% and 68.39% respectively compared to control plants. However, application of KN to NaCl stressed plants enhanced the *Pn* by 82.88%, *A* by 52.97%, *gs* by 147.11% and *E* by 74.54% over NaCl-alone treated plants (Fig 2A–2D).

### Relative water content

NaCl application resulted in a 35.91% reduction in RWC, however, supplementation of KN (NaCl + KN) enhanced the RWC by 17.48% over NaCl treated plants alone (Fig 2E).

### H_2_O_2_, MDA and EL

**Fig 3:**
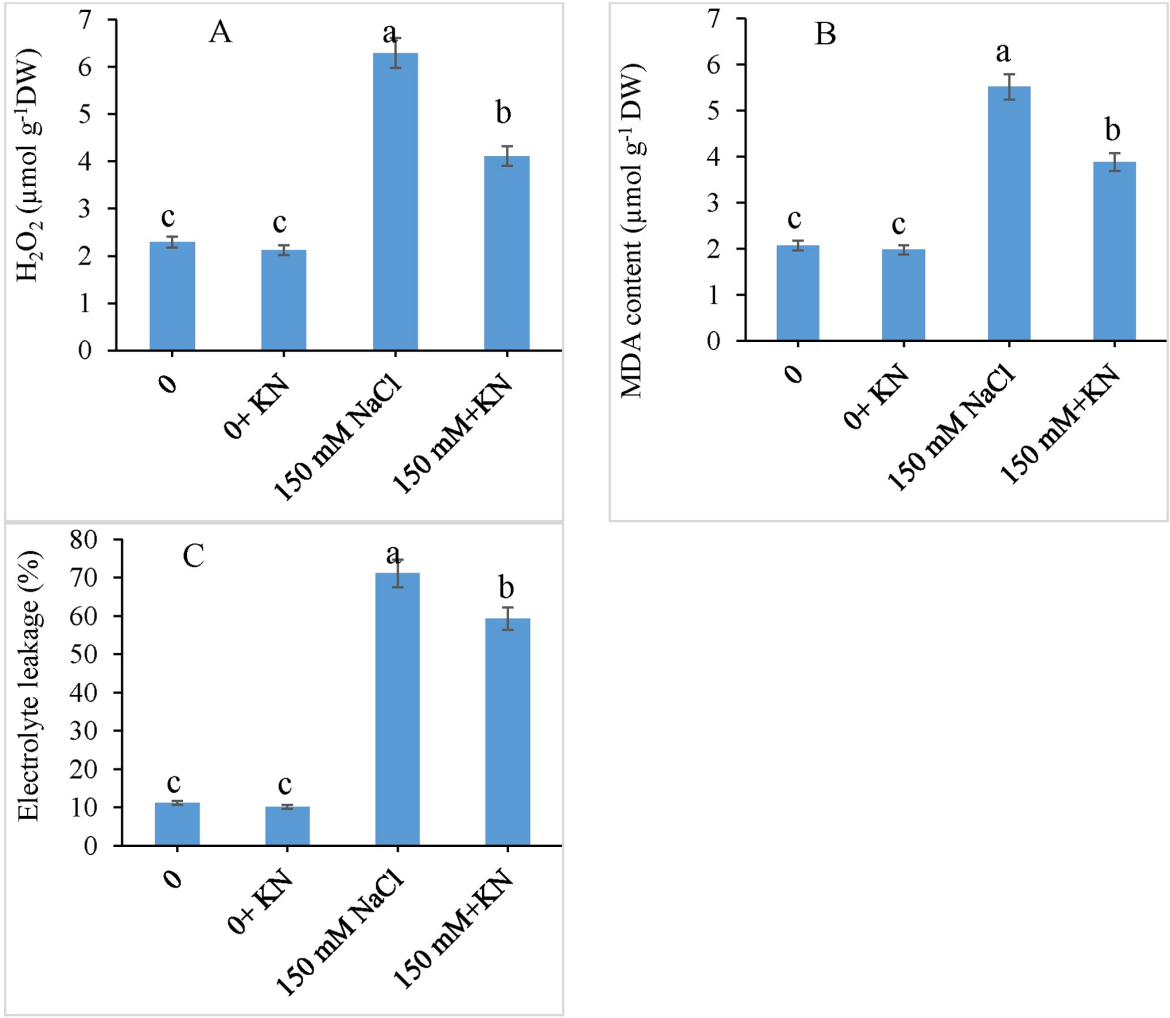
Effect of kinetin (KN) on H_2_O_2_, MDA accumulation and EL in tomato under NaCl toxicity. Data presented are the means ± SE (n = 3). Different letters indicate significant difference at P ≤ 0.05.

Salt (NaCl) stress caused an increment in the production of hydrogen peroxide (H_2_O_2_), lipid peroxidation (measured in terms of malondialdehyde (MDA) formation), and electrolyte leakage by 2.74 fold, 2.66 fold and 6.37 fold respectively over control plants. However, application of KN to NaCl treated plants decreased the H_2_O_2_ by 1.53 fold, MDA by 1.42 fold and EL by 1.19 fold over NaCl treated plants alone (Fig 3A–3C).

### Antioxidant enzymes and ascorbate-glutathione cycle

Relative to the control, NaCl-stressed plants exhibited an increase of 61.43% in SOD, 74.34% in CAT, and 77.79% in GST activities, which were further enhanced by 16.99%, 29.60%, and 25.29% respectively, by the application of KN compared with those treated with NaCl-alone.

**Fig 4:**
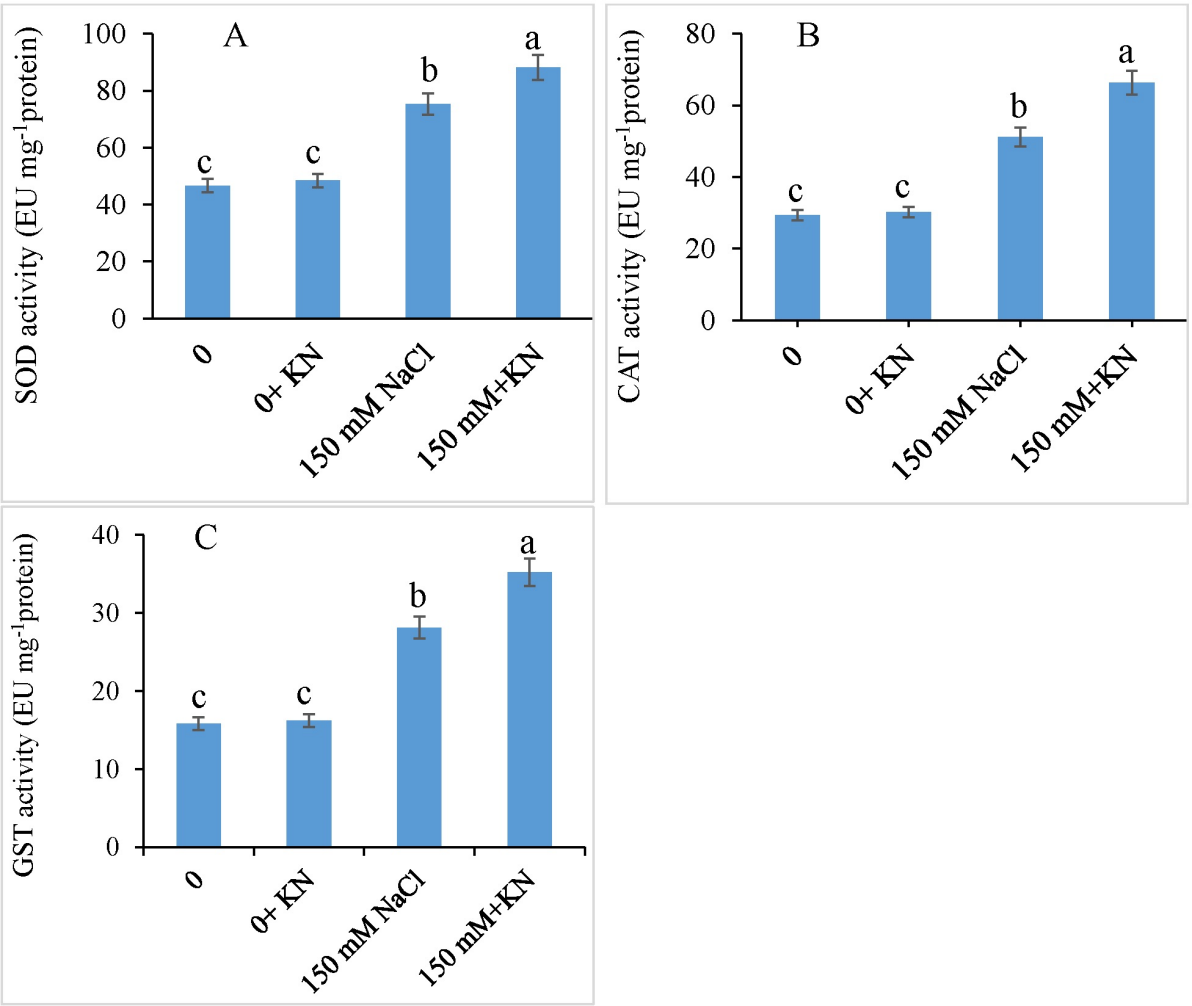
Effect of kinetin (KN) on activity of SOD, CAT and GST in tomato under NaCl toxicity. Data presented are the means ± SE (n = 3). Different letters indicate significant difference at P ≤ 0.05.

**Fig 5:**
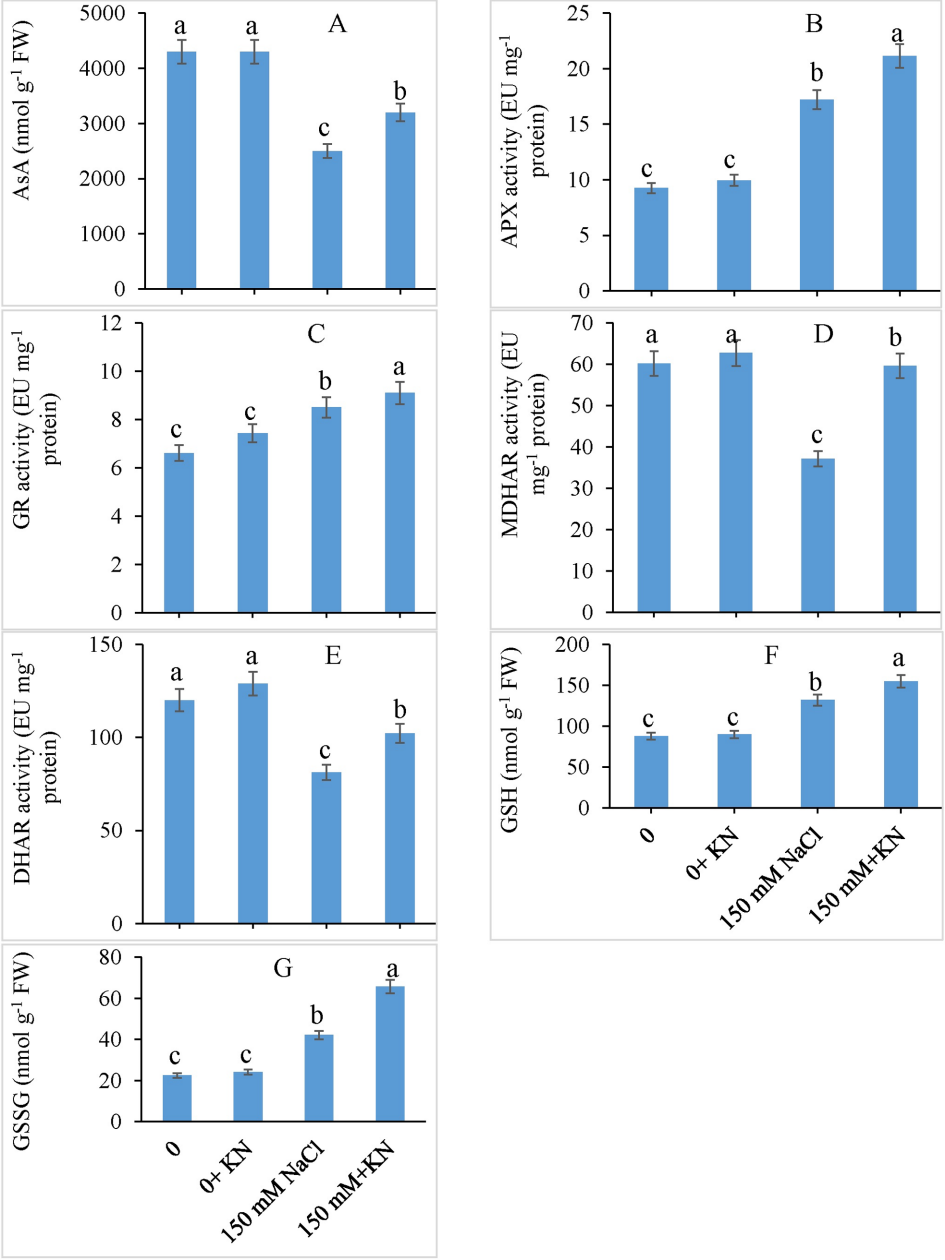
Effect of kinetin (KN) on ascorbate-glutathione cycle in tomato under NaCl toxicity. Data presented are the means ± SE (n = 3). Different letters indicate significant difference at P ≤ 0.05.

The effect of NaCl and KN on ascorbate-glutathione cycle is presented in Fig 4A–4C. The content of ascorbic acid was observed to be decreased by 41.86% due to NaCl stress, however, KN application mitigated this reduction by causing a slight increase of 28.00% compared with that in the NaCl-treated plants alone (Fig 5A). The NaCl enhanced the APX and GR activity by 86.05% and 28.74% respectively over control, however. External supplementation of KN, further enhanced the APX activity by 22.89% and Gr activity by 6.93% compared to NaCl treated plants alone (Fig 5B and 5C). Although NaCl reduced the activity of MDHAR (38.30%) and DHAR (32.31%) over control, KN (NaCl + KN) was observed to reinforce their activity by 60.56% and 25.74% respectively over NaCl stressed plants (Fig 5D and 5E). Relative to the control, NaCl treated plants exhibited an increase of 50.00% and 87.07% in GSH and GSSG, respectively. Compared with the NaCl-stressed plants, NaCl + KN-treated plants exhibited an increase of 17.42% in GSH and 56.04% in GSSG content (Fig 5F and 5G).

### Glyoxalase system

**Fig 6:**
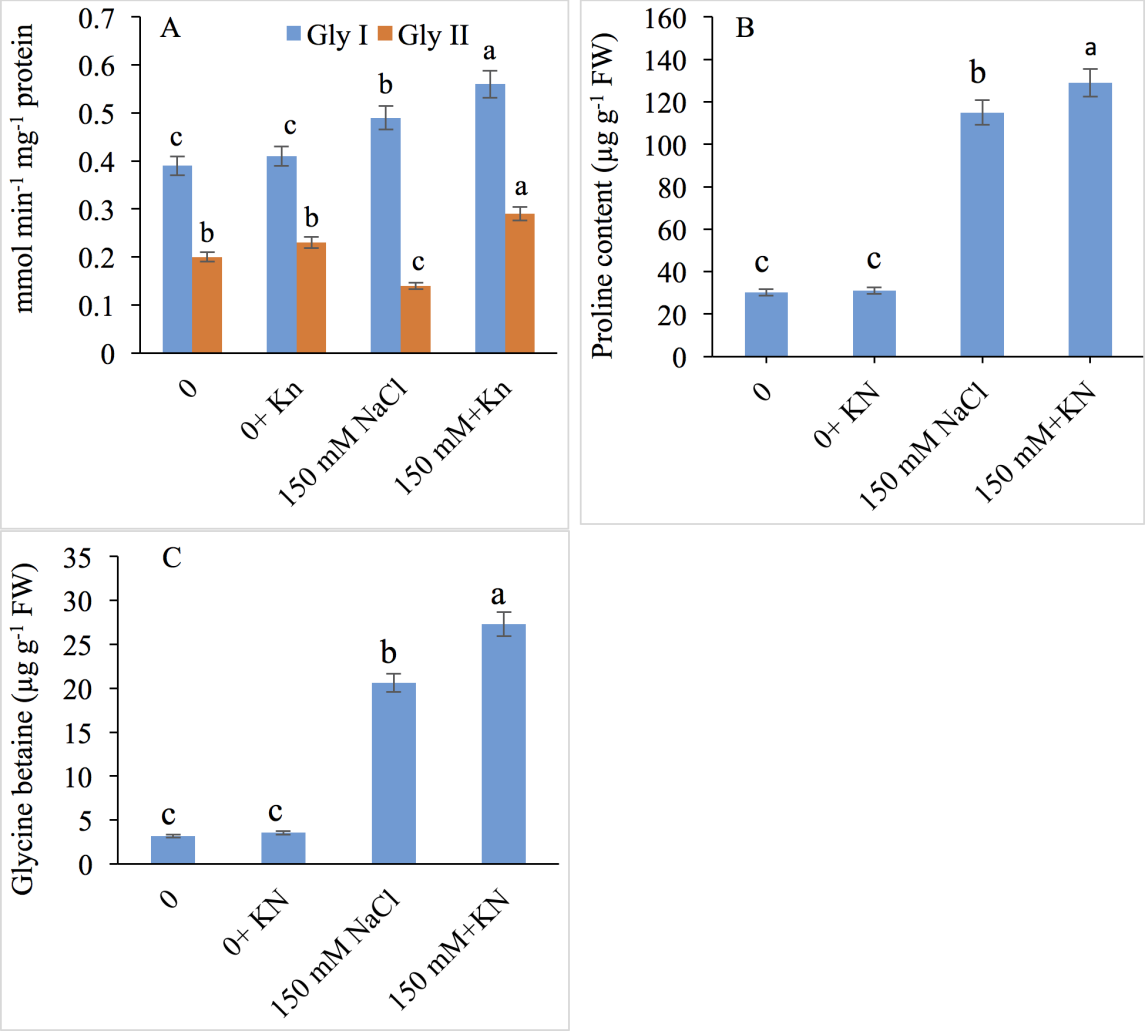
Effect of kinetin (Kn) on Gly I and Gly II, proline and glycine betaine accumulation in tomato under NaCl toxicity. Data presented are the means ± SE (n = 3). Different letters indicate significant difference at P ≤ 0.05.

NaCl stress increased the activity of glyoxylase I (20.40%) while it decreased that of glyoxylase II (25.64%) activity over control. Application of KN (10 μM) to NaCl stressed plants further stimulated the activity by 14.28% (glyoxylase I) and 107% (glyoxylase II) over those of NaCl stressed counterparts (Fig 6A).

### Total phenols and flavonoids

NaCl treatment increased total phenols (78.61%) and decreased flavonoids (64.99%) over control plants. Contrary to this, application of KN to NaCl stressed plants further increased the contents of total phenols by 26.18% and ameliorated the decline in flavonoids by 51.38% over NaCl stressed plants alone (S1 Table).

### Proline and glycine betaine

In NaCl-treated plants, free proline and glycine betaine were observed to increase by 3.81 fold and 6.50 fold, respectively, compared with the control; however, exogenous application of KN (NaCl + KN) further increased the contents of free proline and glycine betaine by 1.12 fold and 1.32 fold, respectively, compared with salt stressed plants alone (Fig 6B and 6C).

### Tissue Na, K and Ca

Salt treatment caused a 71.80% and 44.54% decline in the uptake of K^+^ and Ca^2+^, respectively. KN-supplemented salt-treated plants exhibited a reduction of 48.81% in Na^+^ accumulation, which was reflected in a significant decline in the Na^+^/K^+^ ratio compared to that in the NaCl-treated plants (Table 3).

**Table 3:** Effect of kinetin (Kn) on Na, K and Ca (mg g^-1^ DW) uptake in tomato under NaCl toxicity. Data presented are the means ± SE (n = 3). Different letters indicate significant difference at P ≤ 0.05.

Treatments	Na^+^	K^+^	Na/K ratio	Ca^2+^
**0**	15.27±0.81c	31.11±1.39b	0.49±0.009c	5.32±0.032b
**0+ Kn**	14.19±0.76d	34.12±1.42a	0.47±0.008c	6.11±0.035a
**150 mM NaCl**	37.61±1.44a	8.77±0.66d	4.28±0.022a	2.95±0.019d
**150 mM +Kn**	19.25±0.96b	15.32±0.82c	1.25±0.011b	4.77±0.028c

## Discussion

Salt stress reduced the length and biomass accumulation of *S*. *lycopersicum*, whereas KN maintained and stimulated its growth under both normal and stress conditions. Salinity stress has been reported to hamper the export of cytokinins such as KN from the root to the shoot [43], thereby decreasing the shoot KN concentration required for normal growth and development [44]. In the present study, exogenously applied KN may have maintained cell division at an optimal level. Our results of amelioration of the salinity-induced decline in length and biomass by KN, are consistent with the findings of Iqbal and Ashraf [45] and Kaya et al. [46] who also reported cytokinin-induced growth promotion in wheat and maize respectively. In *Solanum melongena*, Singh and Prasad [21] have also demonstrated enhanced growth and mitigation of metal stress due to exogenous application of KN. The salinity induced reduction in growth was correlated with a significant reduction in the synthesis of chlorophyll pigments and decrease in membrane damage. Earlier, Dhindsa et al. [47] and Chen et al. [48], have demonstrated a significant effect of stress-triggered membrane leakage on chlorophyll biosynthesis. The results for salt-induced reduced chlorophyll contents corroborate with those of Rasool et al. [49] in *Cicer arietinum* and Ahanger and Agarwal [3] in *Triticum aestivum*. Higher concentrations of NaCl have been reported to reduce pigment synthesis and pigment protein complex functioning [50] in addition to reducing the *de novo* synthesis of proteins and associated chlorophyll components [51]. For example, Das et al. [52] have reported improved yield in mulberry after KN mediated enhancement in chlorophyll synthesis. Enough cytokinin concentration triggers etioplast-chloroplast transitions accompanied by the greater appearance of the metabolites implicated in the chlorophyll tetrapyrrole biosynthesis pathway [53], which probably is regulated by the effect of cytokinin on the expression of key chlorophyll biosynthesis genes including *HEMA1*, *GUN4*, *GUN5*, and *CHLM* [54].

Photosynthetic efficiency was reduced by NaCl-mediated decline in the chlorophyll fluorescence parameters, and KN proved ameliorative by increasing *F*v/*F*m, ϕPSII, ϕ_exc_, qP and decreasing NPQ. Stresses are believed to damage the antenna pigments thereby restricting the transfer of electrons from PSII to PSI [55]. Our results suggest that KN-mediated improved efficiency may largely depend on the protected functioning of the photosynthetic light reactions synchronized functionally with the enzymes of PSII and PSI. Moreover, functioning of the photosystems is coupled to generation of trans-thylakoid proton gradient creating pH difference for regulating transfer of electrons to PSI, and this transfer has been reported under the direct control of proton gradient regulation-5 (PGR5) protein [56]. It can be inferred that greater PSII activity in KN supplemented plants may have prevented the formation of singlet oxygen thereby protecting the chloroplast structure from the oxidative damage. Application of KN was reported to protect the pigment protein complex from high temperatures in *Zea mays* [57]. KN also nullified the effect of waterlogging [58] on the Rubisco and Hills activity, and of salinity [16] on leaf area, Hill’s reaction and chloroplast structure resulting in a significant improvement in net carboxylation efficiency. KN application to *Solanum lycopersicum* was found to be effective in maintaining and protecting CO_2_ assimilation rate, *g*_*s*_ and *E* under normal as well as NaCl treated conditions, thereby leading to better growth performance. Application of phytohormones has been observed to assuage the damaging effects of NaCl on the photosynthetic efficiency significantly [59], and thus future research in this direction can be worthwhile. It has been opined that phytohormones can improve the synthesis of proteins and polysaccharides involved in photosynthetic regulation [60]. However, in the present study, KN-mediated synthesis of redox components may have contributed to photoprotection. In another study, KN was reported to trigger the synthesis of carotenoids, which protected the photosynthetic apparatus from excess ROS by up-regulating the activities of pigment-synthesizing enzymes coupled with reduction in the degrading enzymes, the activity of which is otherwise reported to be increased due to stress [61].

Reduced H_2_O_2_ accumulation, lipid peroxidation and electrolyte leakage in KN treated plants under saline stress were observed. Such a protective role of KN has been reported earlier also [62]. Siddiqui et al. [63] reported the amelioration of storage-induced degradation of cauliflower due to treatment with 6-benzylaminopurine. Salinity triggered membrane leakage and hence lipid peroxidation can be mitigated by KN supply [8,49,64]. However, KN mediated enhanced membrane integrity can be ascribed to its involvement in maintenance of tissue water content, the antioxidant system and peroxidation rates, which are greatly affected by stresses.

Application of KN promoted the activities of antioxidant enzymes and enhanced the contents of AsA and glutathione (GSH and GSSG), thereby imparting protection to *S*. *lycopersicum* plants against NaCl-induced oxidative stress. Cytokinin-induced up-regulation of antioxidant system has been reported by Wu et al. [20], Wang et al. [65]; and Siddiqui et al. [63]. During antioxidant dismutation of ROS, SOD eliminates superoxide radicals with CAT and APX carrying the further dismutation. KN-induced up-regulation of SOD may cause modulation of the substrate superoxide and H_2_O_2_, thereby reducing the formation of more toxic hydroxyl (OH^-^) radicals [21]. Wang et al. [65] have demonstrated cytokinin-induced temperature stress tolerance in bentgrass through induction of the activities of antioxidant enzymes. The KN-induced accumulation of AsA, GSH and GSSG can protect NaCl-stressed tomato plants from ROS-induced damage. APX, GR, DHAR, MDHAR, AsA, and GSH are the components of the ROS scavenging pathway (the ascorbate-glutathione cycle) and KN-induced up-regulation of these components strengthens the tolerance strategies against any possible oxidative damage. For example, salt stressed *Solanum melongena* treated with exogenous cytokinin exhibited reduced ROS accumulation and greater protection to photosynthetic pathways leading to better yield productivity. Under high salinity, Mittal et al. [66] have reported enhanced growth in *Brassica juncea* accompanied by increased CAT activity. H_2_O_2_ produced by the SOD activity can be eliminated by either CAT in the cytoplasm or APX in the ascorbate–glutathione cycle, which comprises a series of redox reactions, involving the active engagement of ascorbate, glutathione, and NADPH [67]. APX plays an important role in scavenging H_2_O_2_ in chloroplasts and the cytosol thereby preventing its diffusion to other organelles to avert the damage. Optimal functioning of the AsA-GSH pathway due to exogenous KN maintained the redox components including AsA and glutathione, thus reducing the oxidative stress effects of salinity. The increased activity of antioxidants is correlated with the enhanced tolerance of plants to stress [3,68,69]. Up-regulation of the activities of DHAR and MDHAR due to KN application led to the optimization of AsA and glutathione integrating the functioning of other enzymatic components including APX and GR with the non-enzymatic components, therefore causing an overall effect on the H_2_O_2_ neutralization and the availability of NADP for greater protection of electron transport chain [70]. In the present study, protection to the photosynthetic electron transport chain by KN could have been due to precise regulation of NADP^+^/NADPH ratio, which prevents the flow of electrons to molecular oxygen subsequently restricting the generation of superoxide radical [20]. Cortleven et al. [71] have demonstrated that *Arabidopsis* mutants deficient in cytokinin production display greater intensity of stress-induced photodamage by exhibiting a declined D1-repair, which was found correlated with the reduced synthesis of AsA and glutathione. The present investigation also infers the protective role of KN for photosynthetic system through the up-regulation of antioxidant system. However, further studies on investigating the expression patterns of antioxidant molecules as affected by KN application are required.

Higher concentrations of GSH in KN treated plants may have contributed significantly to the maintenance of the glyoxylase system for elimination of methylglyoxal, which may decline the chances of any serious genotoxic effect [72]. Glyoxylase I and II form the key enzymatic components of the glyoxylase system, and KN-induced up-regulation in their activities, accompanied by the increased GSH, may have led to exploit the beneficial effects of methylglyoxal like crosstalk with important signaling molecules like Ca, ROS and ABA [73]. Reports discussing the involvement of cytokinins in optimization of methylglyoxal are very scanty, and thus further analysis at proteomic or genomic levels shall be handy in further consolidations of any possible role in methylglyoxal metabolism. Glyoxylase I uses GSH as a cofactor in converting methylglyoxal to *S*-D-lactoylglutathione, and glyoxylase II generates GSH thereby contributing to redox homeostasis and protection against toxic species [74]. Transgenic plants exhibiting increased expression of glyoxylase I exhibit apparent improvement in stress tolerance via enhanced production of GSH [75]. Therefore, it could be inferred that enhancing endogenous GSH by the application of KN and the functioning of methylglyoxal scavenging system can improve tolerance potential of plants. For example, in salt stressed rice, Rahman et al. [12] have also demonstrated up-regulation of the glyoxylase system. Enhanced activity of the glyoxylase system due to exogenous application of KN may have protected the electron transport system by preventing damage to chloroplast and mitochondrial ultrastructures [72].

Antioxidant system was reinforced by the greater synthesis of phenols and flavonoids in the KN-treated plants (S1 Table). It has been reported that greater production of phenolics regulate plant developmental processes like lignin and pigment biosynthesis, thereby providing structural integrity and scaffolding support to the plants [76]. KN-induced improvement in accumulation of phenols enhances the possibility of crosstalk with the signaling molecules like nitric oxide for improving the efficiency of assimilatory pathways like nitrogen metabolism [77]. Ahanger and Agarwal [3] demonstrated that greater accumulation of phenolic compounds imparts better ROS scavenging potential, resulting in greater protection of membranes and nucleic acids, and at times particularly under stress can contribute to osmoregulation [69]. In the present study KN-mediated accumulation of phenols may have contributed to strengthening of cell wall structures and prevention of oxidative damage to membrane lipids and proteins by modulating their peroxidation kinetics [78]. For example, presence of cytokinins greatly affected the synthesis of phenols in *Tulbaghia simmleri* [79].

In our investigation, salt treatment triggered the accumulation of proline and glycine betaine, and further enhancement resulted due to exogenous application of KN. In salt-stressed *Morus alba*, Harinasut et al. [80] have also reported a manifold increase in the accumulation of proline. Accumulation of proline is attributable to the up-regulation of proline synthesizing enzymes and subsequent down-regulation of the catabolizing enzymes, or alternatively can be due to its decreased incorporation into proteins [8,81,82]. KN-induced enhancement in the accumulation of proline and glycine betaine to some extent might have helped tomato plants to avert the salinity-induced decline in tissue RWC. Enhanced RWC due to exogenous application of cytokinins in copper and zinc treated *Lupinus termis* [83] and Pb-stressed safflower [62] plants has been reported, which may be attributed to increased hydraulic conductivity which is however significantly affected by stresses [84]. Proline and glycine betaine maintain water balance of plants thereby minimizing the deleterious effects of stresses on metabolism [6] particularly by protecting protein turnover, enzyme activities and the expression of stress-protective proteins [3,5,69,85]. KN mediated increased proline and glycine betaine synthesis under normal and NaCl-stressed conditions is an indicative of its protective role for metabolically important pathways by bringing osmoregulation, though the role of exogenously applied cytokinin in regulating osmotic balance is yet inconclusive (S1 Table). Therefore, KN-mediated increase in tissue water content resulted in maintained cell wall extensibility and cell division, which cause increased growth and biomass accumulation.

Exposure of *S*. *lycopersicum* plants to NaCl stress impeded the uptake of essential mineral elements such as K and Ca. Excess uptake of Na^+^ intensifies its antagonistic relationship with important ions, including K^+^. The salinity-induced reduction in ion uptake is in agreement with Garg and Manchanda [86] in *Cajanus cajan*, Kohler et al. [87] in lettuce and Ahmad et al. [88] in *Brassica juncea*. Exogenous application of KN not only prevented Na toxicity by reducing its uptake, but it also caused a significant increase in the uptake of K^+^ reflecting in declined Na^+^/K^+^ ratio, an important attribute for regulating major cellular pathways and protecting them from Na toxicity [5,88]. Reduced Na^+^/K^+^ ratio results from the efficient exclusion of toxic ions leads to maintenance of osmotic potential and hence stress tolerance [89], and tissue Ca^2+^ concentration facilitates K^+^/Na^+^ selectivity [90]. During NaCl stress, cytosolic Ca^2+^ is maintained at high concentrations, as a consequence of transport from the apoplast and the intracellular compartments [91]. Such transient increases in cytosolic Ca^2+^ due to KN may contribute to initiate the signal transduction pathways that result in enhanced salt tolerance. Sodium chloride (NaCl) competes with Ca^2+^ at membrane binding sites [92]. Foliar application of KN to tomato plants might have mitigated the reduction in K and Ca uptake caused by salinity, resulting in increased K^+^/Na^+^ and Ca^2+^/Na^+^ ratios and plant performance by regulating the enzyme activity, membrane stability and stomatal functioning[2].

## Conclusions

On the basis of the results of this study, it can be concluded that exogenously applied KN resulted in enhanced growth performance of *S*. *lycopersicum* plants under NaCl stress by up-regulating antioxidant metabolism and osmolyte accumulation. Reduced ROS production in parallel with reduced lipid peroxidation in KN-supplemented plants resulted in the maintenance of cellular functioning, greater photoprotection and mineral uptake, which caused enhanced protection of photosynthetic pigments.KN-mediated growth promotion under stressed conditions may be ascribed to strengthening of the ROS and methylglyoxal detoxifying systems by way of integration between different signaling components. Overall, these observations indicate the suitability of foliar application of KN for exploiting the genetic potential of *S*. *lycopersicum* under NaCl stress.

## Supporting information

S1 Table Effect of kinetin (KN) on total phenol and flavonoids in tomato under NaCl toxicity. Data presented are the means ± SE (n = 3). Different letters indicate significant difference at P ≤ 0.05.
(DOCX)

S1 Dataset individual level data. Effect of Kn on growth, biomass yield, biochemical attributes, lipid peroxidation, antioxidants and secondary metabolites in tomato Under NaCl stress.
(XLSX)
