# Supplementary material for: Potential of exogenously sourced kinetin in protecting Solanum lycopersicum from NaCl-induced oxidative stress through up-regulation of the antioxidant system, ascorbate-glutathione cycle and glyoxalase system
Source: PLoS One. 2018 Sep 4;13(9):e0202175. doi: 10.1371/journal.pone.0202175 (PMC6122799; doi:10.1371/journal.pone.0202175)
Supplement: S1 Table — Data presented are the means ± SE (n = 3). Different letters indicate significant difference at P ≤ 0.05. (DOCX) [file pone.0202175.s001.docx]

| **Treatments** | **Total phenols (mg GAE g^-1^ extract)** | **Flavonoid content (mg catechin g^-1^ extract)** |
| --- | --- | --- |
| Control (0) | 7.34±0.52d | 18.51±1.07b |
| 0 + Kn | 8.55±0.61c | 21.96±1.13a |
| 150 mM NaCl | 13.11±0.79b | 6.48±0.45d |
| 150 mM NaCl + Kn | 17.76±0.99a | 9.81±0.66c |

**Table S1**: Effect of Kinetin (KN) on total phenol and flavonoids in tomato under NaCl toxicity. Data presented are the means ± SE (n = 3). Different letters indicate significant difference at P ≤ 0.05.
